# Editorial

**Published:** 1903-11-15

**Authors:** 


### EDITORIAL.

We give up much of our space this month to record memorial tributes to our friend and associate for many years. It seems peculiarly fitting that this journal to which he gave so many years of service, and through which he did so much to help build up the calling to which he gave himself into one of the recognized professions of the world, should make a suitable record of the main facts of his professional life. He was firmly convinced that through the dental society and the journal literature more could be done to consolidate and elevate professional ideals than in any other way. He did all he could by personal attendance and contributions to keep up a general interest in society work, and he felt that a printed record of the proceedings of dental societies was a valuable means of disseminating the work of the society. He therefore was willing to sacrifice his time and means that this journal should perform this mission to the best of its ability. It was this thought that inspired him with the determination to maintian it in times when it was a great burden to him to do so. For nearly fifty years he was connected with this journal, most of that time as editor and proprietor. In an editorial in the January, 1900, REGISTER he states his feelings at severing his long connection with this work in characteristic words, which we quote: "It is not without regret, loneliness and a feeling of homesickness that I vacate the editorial chair. I shall greatly miss the opportunity of counseling with my professional brothers from whom it has been my happy privilege to receive the kindest consideration, which has made pleasant my pathway and indeed given a zest to my professional life that can never be forgotten" It is impossible to place any estimate on the value of such a life, devoted as it was to the highest interest of his profession. He thought and worked with a singleness of purpose and tenacity that few men would sustain through so many years, and it was not possible to detect in his last days any indication of a desire or purpose to relax, but on the contrary he was planning to undertake new work in connection with his college teaching. As we review his life we cannot see how any man could have done more. In fact we can't but feel that his life was in a peculiar sense consecrated to his profession. He enjoyed success and was happy when it appeared that advance was being made; but if adversity came to his plans he was, so far as any one about him could determine, just as cheerful as though he had gained a victory. How often have we heard him, when things did not seem to be going as smoothly as he wished, hum a little song with the refrain, "There's a good time coming boys."

How we shall miss him! We do not yet realize that he has gone. We have many personal memories that are precious and his spirit has so shaped our lives that he still lives for us, and it may be that the momentum of his life may serve to speed us onward toward those nobler conceptions which made his a life worth living and one to imitate. He has gone on to enjoy the blessings of eternity with his early associates, most of whom have long preceded him, but his work and their's remain, some unfinished, andf or us an obligation, much more a precious heritage.

—✦—

THE meeting of the Institute of Dental Pedagogics and the American Society of Orthodontists will be held at Buffalo beginning the last week of December. The Pedagogic meetings will be held on Monday, Tuesday and Wednesday, and the Orthodontia meetings on Thursday, Friday and Saturday. These meetings will be interesting to practitioners as well as teachers. Both societies occupy unique places. In the Pedagogic Society no business matters are discussed, but the whole time is given to the discussion of teaching methods and partakes very often of a kind of experience meeting. We know of no more valuable professional gathering for teachers during the whole year. And many practitioners after attending these meetings have been pleased to say they have never attended more interesting and helpful meetings. There are no executive sessions, and any one who chooses to do so may attend. We trust there may be a large gathering. We have never attended a meeting of the Orthodontists, but are looking forward to one week of solid pleasure and profit. It will be a rare chance for the members of the profession to take a vacation and get into touch with what is going on among the thinkers of the profession. It would be a good scheme if the members of the various State Examining Boards could attend and get an idea of what the college teachers are striving to accomplish. Perhaps they would find out that the college men are after all not such a bad lot.

